# Assessment of the frequency of coughing and sneezing triggered by nasopharyngeal swabbing in the pandemic setting

**DOI:** 10.1038/s41598-022-14755-0

**Published:** 2022-06-27

**Authors:** Cosmin Andrei Cismaru, Sergiu Chira, Gabriel Laurentiu Cismaru, Andreea Mihaela Nutu, Mihai Gheorghe Netea, Ioana Berindan-Neagoe

**Affiliations:** 1 The Research Center for Functional Genomics, Biomedicine and Translational Medicine, “Iuliu Hatieganu” University of Medicine and Pharmacy, Cluj-Napoca, Romania; 2 Fifth Department of Internal Medicine, Cardiology Rehabilitation, “Iuliu Hatieganu” University of Medicine and Pharmacy, Cluj-Napoca, Romania; 3 Department of Internal Medicine and Radboud Centre for Infectious Diseases, Radboud University Nijmegen Medical Center, 6525 GA, Nijmegen, The Netherlands

**Keywords:** Infectious-disease diagnostics, Virology

## Abstract

A variety of medical procedures are classified as aerosol generating. However there is no consensus on whether some procedures such as nasopharyngeal swabbing can generate aerosols. During specimen collection, the contact of the nasopharyngeal swab with the respiratory mucosa often triggers defense reflexes such as sneezing and coughing, which generate airborne particles. The accumulation and persistence of a viral load from infectious aerosols for hours after their generation can represent a threat for increased spread of infection. Prospective observational cohort study in individuals tested for RT-PCR SARS-CoV-2 from July to October 2020. Participants were evaluated for the prevalence of aerosol generating events (AGEs) triggered by the nasopharyngeal swabbing. We used descriptive statistics to analyze the data set and the chi-square test for AGE comparison between sexes. Among 1239 individuals, we reported 264 in which AGEs were triggered by the specimen collection. 97 individuals tested positive for SARS-CoV-2, of which 20 presented AGEs. There were no significant differences in the occurrence of AGEs by age, but significant differences have been identified between sex and the occurrence of AGEs both in the SARS-CoV-2 negative and SARS-CoV-2 positive individuals. The prevalence of coughing or sneezing triggered by the nasopharyngeal swabbing was high among tested individuals. Testing facilities should ensure adequate availability of personal protective equipment (PPE) for the testing personnel, ensure appropriate ventilation of the rooms, and develop additional strategies to limit the risk of contamination of other participants to the testing session from potentially infectious and persistent aerosols.

## Introduction

**Figure 1:**
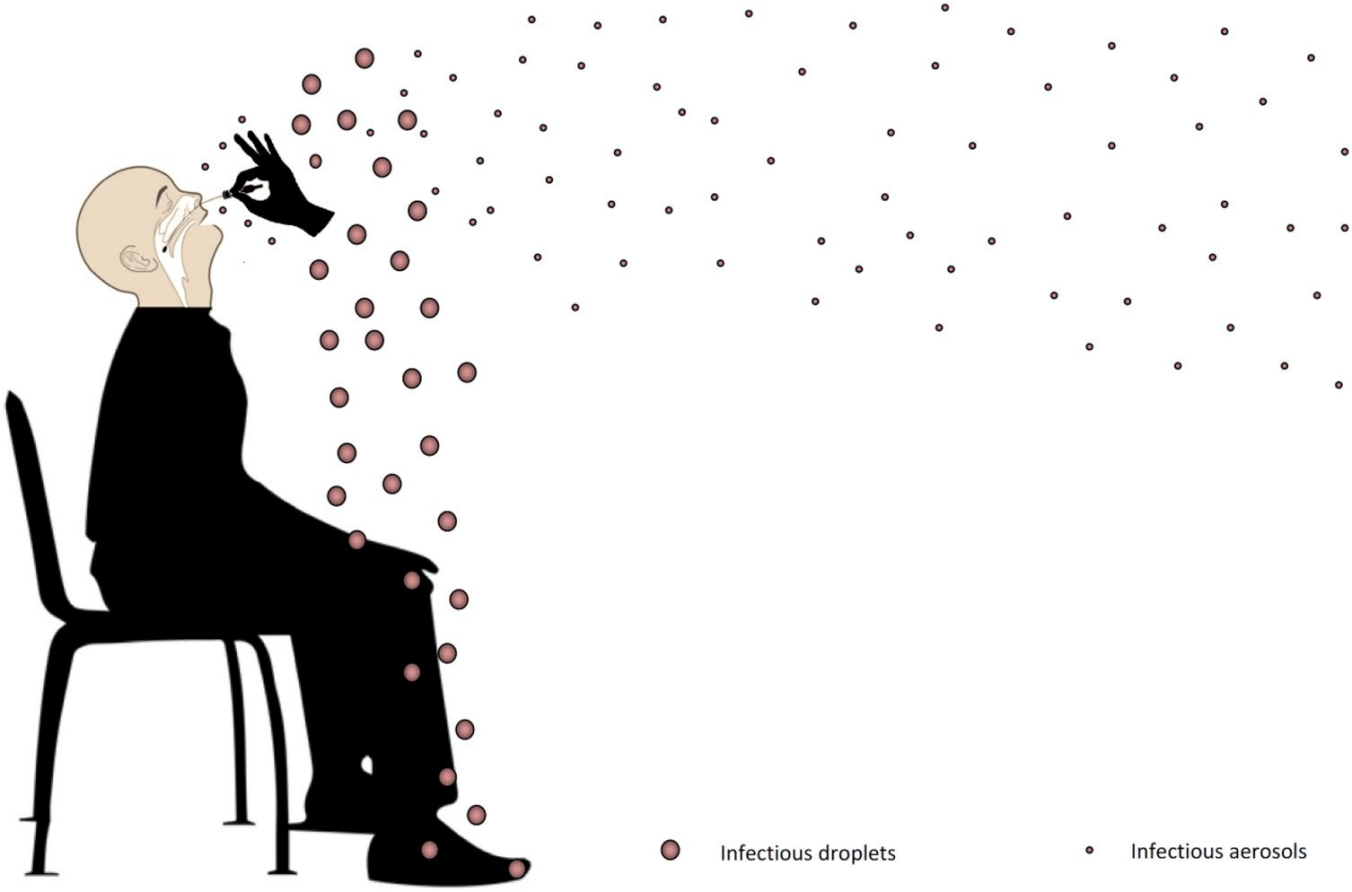
Schematic representation of the generation of Flügge's droplets by AGEs. Specimen collection by nasopharyngeal swabbing can generate reflexes of coughing and sneezing. In an infected person, such airborne particle producing events can lead to virus containing droplets (Flügge's droplets) falling in the proximity of the infected person while some droplets become aerosols by evaporation into droplet nuclei which can linger for many hours in the ambient air of enclosed spaces.

Sneezing, coughing, and even higher amplitude breathing can produce natural water droplets which can enclose a variety of particles from epithelial and immune cells to different infectious agents such as bacteria, fungi and even viruses^1^. Some of the earliest contagion studies from pathogen aerosolization were carried out by Carl Flügge in the late 1800s^2^. Droplets ≤ 5 µm in diameter become airborne and such aerosols can linger in the ambient air and remain pathogenic for many hours after being produced. SARS-CoV-2 was reported to be twice more aerostable than the influenza virus and 4 times more aerostable than filoviruses^3^. The place where the aerosols are generated can dictate whether sufficient viral copies are passed from one host to another to become pathogenic. Droplets and aerosols invisible to the naked eye which are generated by coughing and sneezing have been previously measured by image-capturing systems using classical methods such as high-speed photography and newer approaches using high-vision/high speed computer equipped video recording systems^4,5^. Such airborne particles were shown to be able to carry infectious influenza virions, suggesting similar contagion mechanisms for viruses with a tropism for the respiratory tract^6–9^. In a modeling study of inhaled droplet transport to the nasopharinx, Basu S. estimated that the pathogenic viral load necessary to infect a susceptible host vas approx. 330 virions for SARS-CoV-2^10^. This is 6–9 times smaller than the dose necessary for the infectivity of Influenza A which was estimated at approx. 1950–3000 virions^8^. While outdoor spaces can lead to the dispersion of the viral copies, enclosed spaces can hold up an increased viral load in the air for prolonged periods of time after the occurrence of an aerosol generating event (AGE). This can become a concern in specimen collection units for SARS-CoV-2 testing since the contact of foreign objects (e.g. a flocked swab) with the mucosa of the respiratory tract of a person being tested can trigger defense reflexes such as coughing and sneezing. Addressing the potentially infectious and persistent aerosols resulting from such events prompts for immediate protective measures to be taken in order to prevent the contamination of testing personnel and the following participants to testing. COVID-19 is believed to be transmitted through droplets and aerosols generated when an infected person coughs, sneezes or even talks or exhales. Larger droplets will deposit near the emission point, while smaller droplets evaporate faster in the form of aerosols (< 5 µm in diameter) and linger in the air, drifting and traveling meters or tens of meters in indoor air^11^ (Fig. 1).

The highly contagious SARS-CoV-2 has already spread rapidly on a global scale claiming 3000 times more lives than the previous SARS and MERS coronavirus epidemics combined^12^. The increased capacity of infecting many people at a time is believed to be attributed to the capability of becoming trapped in infectious droplets and aerosols which plays a vital role in contracting susceptible nearby hosts^13^. This raises concerns about the potential persistence in the air, contamination of objects and human-to-human transmission of the virus through the accumulation of a persistent viral load from infectious aerosols in specimen collection rooms. Infectivity assessments with SARS-CoV-2 have reported the virus to remain viable and pathogenic for hours in the ambient air and for days on surfaces after aerosolization^14^. In the context of COVID-19, aerosol generating procedures have been highlighted as requiring a higher grade of personal protective equipment. In the current literature, there is insufficient agreement for the classification of nasopharyngeal swabbing procedure as aerosol generating, prompting for further studies of aerosolization^15^.

The PCR analysis of a nasopharyngeal swab sample is currently the gold standard for COVID-19 testing^16^. The nasopharyngeal mucosa of the respiratory tract is one of the most exposed areas to airborne pathogens. Although a lower detection rate of SARS-CoV-2 is to be expected in the oropharyngeal mucosa due to lower density of ACE2 expression, the extension of the viral tropism to the throat could potentially be explained by the presence of a furin-type cleavage site at the S1–S2 junction in the spike protein that is not present in SARS-CoV, leading to a gain-of-fusion activity that might result in inhanced viral entry as suggested by Wolfel et al.^17^.

The flocked swab specimen collection allows for the coverage of a wide area to be sampled, as it can run from the nasal cavity all the way through the nasopharynx and back to collect mucosal cells on its surface.

While sampling is not traumatic itself, the contact of the respiratory mucosa with the flocked swab can initiate defense reflexes such as sneezing, coughing and lacrimation. While lacrimation poses little risks for contamination^18^, AGEs such as sneezing and coughing can generate airborne viral particles which may persist for long periods in the ambient air and pose a risk for the examiners and the following participants to testing. This raises concerns about the safety of specimen sampling rooms and stresses the need to implement protective measures to avoid the infection of other participants to testing and the contamination of specimens from the exposure of the flocked swab and opened tube to ambient air during the sampling. Our study aimed to assess the extent of coughing and sneezing triggered by nasopharyngeal swabbing for SARS-CoV-2 detection in real-life conditions of testing.

## Methods

### Study design and participants

We performed a prospective observational cohort study between June 2020 and October 2020, evaluating the prevalence of AGEs such as coughing and sneezing during the nasopharyngeal swabbing for RT-PCR SARS-CoV-2 testing. At enrolment, participants consented to use of information for research and agreed to applicable privacy policies and terms of use. Our study was approved by the Ethics Committee of the University of Medicine and Pharmacy “Iuliu Hațieganu” Cluj-Napoca (No. 16761/09.06.2020).

### Procedures

Participants were observed during the nasopharyngeal swabbing for the occurrence of AGEs such as coughing and/or sneezing triggered during the sampling. This was recorded in the database together with the demographical characteristics. COPAN’s^®^ flocked swabs were used for the nasopharyngeal specimen collection which was collected by a certified physician (CCA) for all the samples. The specimens were collected using the method described by Francisco et al.^19^ but with supplementary protective measures for both participants and examiners consisting in using a higher degree of PPE by the personnel and resorting to keeping the facemasks over the mouth of participants during specimen collection to limit aerosol spreading, together with good ventilation of the sampling room, decontamination of air, walls and objects with combined physical and chemical agents after each participant.

### Statistical analysis

All data collected during nasopharyngeal swabbing were introduced into an Excel file which was then exported into SPSS. For descriptive statistics we used: mean, standard and median deviation for the continuous data and for the ordinal or nominal data the frequencies and percentages were used. For AGE comparison between both sexes we used chi-square test. SPSS version 21 was used for all the statistical analysis considering a significant P value of < 0.01.

### Ethics approval

This is an observational non-interventional study. All methods were performed in accordance with the relevant guidelines and regulations. The study was approved by the Ethics Committee of the University of Medicine and Pharmacy “Iuliu Hațieganu” Cluj-Napoca (No. 16761/09.06.2020).

### Consent to participate

Verbal informed consent was obtained prior to performing the data acquisition to limit the exposure period of participants to the testing facility’s environment. Due to the observational non-interventional nature of the study and being a public health issue, a written informed consent was unnecessary according to national regulations. (Lege nr. 677/21.11.2001, art.9a, available at http://legislatie.just.ro/Public/DetaliiDocument/32733). The study methods have been performed in accordance with the Declaration of Helsinki.

## Results

**Figure 2:**
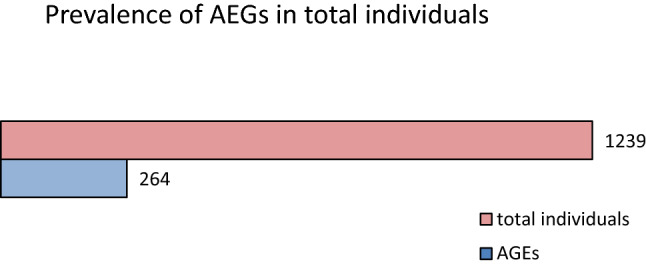
Distribution of AGEs in the study group.

Between July 1 and October 30, 2020, we enrolled 1239 participants to our study, 583 males and 656 females, with ages ranging from 1 to 89 and a median age of 38 [95% confidence interval (39.24–40.77) SD = 13.67]. The prevalence of AGEs triggered by the nasopharyngeal swabbing in our study was 21.23%, consisting of coughing in 13.96% and sneezing in 8.56% of participants (Fig. 2). The occurrence of AGEs was higher in male (26.24%) than in female participants (16.77%) (Fig. 4). The baseline characteristics of the study participants are presented in Table 1.

**Table 1:** Baseline characteristics in all individuals compared with individuals presenting AGEs in the study group.

	Total individuals (%)	Individuals with AGEs (%)
**Sex**		
M	47.05	12.35
F	52.95	8.96
**Age, years**		
< 25	10.25	2.34
25–34	25.59	5.73
35–44	29.06	6.46
45–54	18.40	3.39
55–64	12.75	2.82
≥ 65	3.95	0.56
**SARS-CoV-2**		
Positive	7.83	1.62
Negative	92.17	19.61

**Figure 3:**
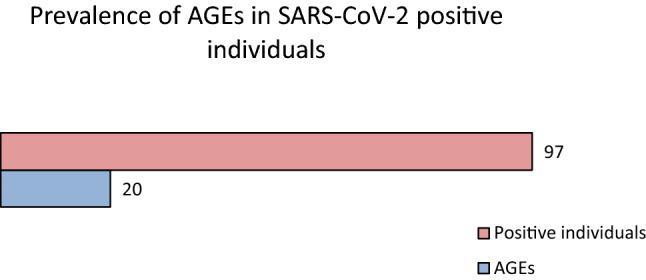
Distribution of AGEs in SARS-CoV-2 positive individuals.

In this cohort we recorded 97 positive participants in the RT-PCR SARS-CoV2 analysis, 53 males and 44 females. The prevalence of triggered AGEs among the positive participants was 20.61% consisting of coughing 15.46% and sneezing in 5.15% (Fig. 3).

There were no significant differences between SARS-CoV-2 positive and SARS-CoV-2 negative participants in the occurrence of coughing and sneezing (p = 0.763).

**Figure 4:**
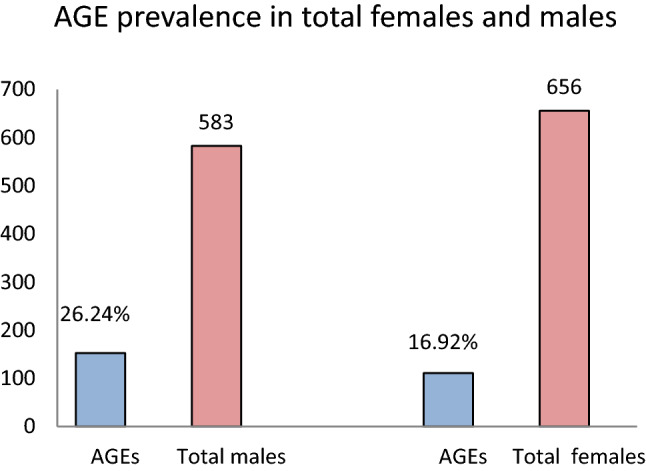
Distribution of AGEs by sex in all individuals.

**Figure 5:**
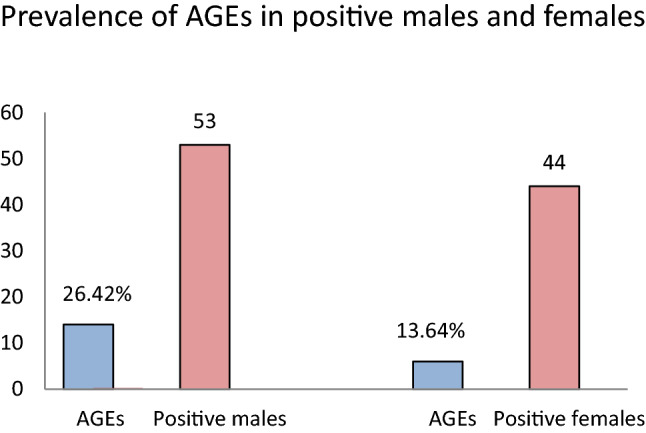
Distribution of AGEs by sex in SARS-CoV-2 positive individuals.

Comparing the two sexes, the prevalence of AGEs in men was higher than in women during the nasopharyngeal swabbing (p < 0.01), both in the SARS-CoV-2 positive and SARS-CoV-2 negative groups (Figs. 4, 5).

## Discussion

Our results show that the nasopharyngeal swab specimen collection is associated with a high occurrence of defense reflexes of sneezing and coughing triggered by the contact of the nasopharyngeal mucosa with the swab. Although a higher occurrence rate of coughing versus sneezing was observed in our study, the body’s choice for the type of defense reflex (coughing or sneezing) triggered by the contact of a foreign body with the respiratory mucosa is mostly dictated by which nerve endings become stimulated first—trigeminal for sneezing or vague for coughing^20^. In a case report, Tsunoda et al.^21^ discuss the importance of avoiding the contact of the swab with the inferior turbinate to prevent induced sneezing. Mechanical stimulation of nasal nerve endings may only partly be responsible for the induction of sneezing and coughing. Wang et al.^22^ discuss in a similar analysis that patients may become uneasy and uncomfortable during swab sampling, coughing, sneezing, and vomiting as a reaction, which might result in droplets, putting medical personnel at risk of exposure.

Whether breathing only by the mask-covered mouth during the nasopharyngeal swabbing can influence the type of evoked defense reflex needs further investigation. Here we have shown that AGEs have occurred in both uninfected and infected persons, being triggered in more than 1 in 5 participants to nasopharyngeal swabbing. This can become a concern when many infected participants show up to testing as it creates the conditions for airborne viral spreading. The prevalence of sneezing and coughing in our study was 13.96% for cough and 8.56% for sneeze and the SARS-CoV-2 negative individuals were used as the most important controls. A similar analysis within the Wang et al. 2020 study, found higher percentages of AEGs on a smaller cohort of 103 patients: 23 out of 103 (22.3%) presented cough and 18 out of 102 (17.5%) presented sneeze during nasopharyngeal swabbing. However, our cohort had 1239 participants compared to 103 in Wang et al. 2020 study^22^.

Depending on the clinical context, the balance between diagnostic advantages and labor safety is likely to change. Lower respiratory aspirates, notwithstanding their dangers, may be required in groups such as hospitalized patients with more severe illness and a strong suspicion of disease despite negative nasopharyngeal swab tests. Individuals at reduced risk, on the other hand, may benefit from less invasive nasopharyngeal examinations^23^.

Less invasive sample collection methods sharing comparable performance could be regarded as alternative measures to reduce droplet and aerosol generation from coughing and sneezing, especially in saliva testing. However, current evidence shows that nasopharyngeal swabs are the gold standard when compared to other specimen types (saliva, oropharyngeal swabs, nasal swabs)^24^.

The human factor analysis is an essential aspect of this kind of study. By having the same medical personnel taking swabs throughout the study, our approach focused on patient safety, constant vigilance and an anticipatory system design, while reducing examiner variability to maximum^25^.

As with all data collection processes, the analysis has both strengths and limitations. Our study addressed the potential contamination risks from dealing with dangerous pathogens which can become airborne and persist for long periods of time in the ambient air from nasopharyngeal swabbing. To our knowledge the study included the biggest cohort in the COVID-19 literature for the study of the prevalence of sneezing and coughing triggered by the nasopharyngeal swabbing in a real world setting and It’s outcomes are invaluable for first-line healthcare workers facing such viral spread. However, some limitations of this study of aerosolization still remain unaddressed requiring further investigations and these include (a) quantification of the aerosols generated by sneezing and coughing triggered by nasopharyngeal swabbing, (b) assessments of the viral load in the resulting aerosols, (c) assessments of the exact risk of contamination from the resulting aerosols both for patients and testing personnel and for the samples being taken and (d) assessments on how long the aerosols generated this way remain pathogenic in the ambient air and on fomites. Addressing these aspects may represent a close perspective for the current COVID-19 pandemic and may provide useful insights for tackling epidemics with other airborne viruses. COVID-19 literatures is new and constantly emerging and recommendations need to be adapted to the most recent findings. The recommendations of Marty ***et al. 2020 on how to obtain a nasopharyngeal swab which were released at the beginning of COVID-19 pandemic include only limited level of PPE for the examiner and no level of protection against potentially infectious and persistent aerosols for the person being tested^19^. Given the prevalence of AGEs triggered by the nasopharyngeal swabbing, the right choice of PPE can make the difference between being safely covered or dangerously exposed. The results of our study suggest the need of a higher grade of personal protective equipment for the examiner and additional safety measures to protect the person being tested from potentially infectious and persistent aerosols in the testing room.

### Strategies of lowering the odds of contamination from an AGE

#### Providing adequate ventilation and volume of air

Adequate ventilation to the sampling room could ensure that the viral load in the ambient air will stay at low levels from air exchanges with the exterior. A natural ventilation is preferred against mechanical ventilation systems since air recirculation devices can accommodate and potentially mobilize viral particles in the air from exhaust outlets^26^.

#### Using washable surfaces

A decontamination of surfaces should be achieved after the occurrence of an AGE before the next participant is invited in. However, this can be done if the sampling room is equipped with readily washable surfaces, on which the decontaminants can be effectively applied. Since airborne viruses can become embedded in fabrics, the textile covering of chairs, table or windows and textile walls such as the ones used in military tents should be avoided.

#### Keeping the facemasks over participant’s mouth

**Figure 6:**
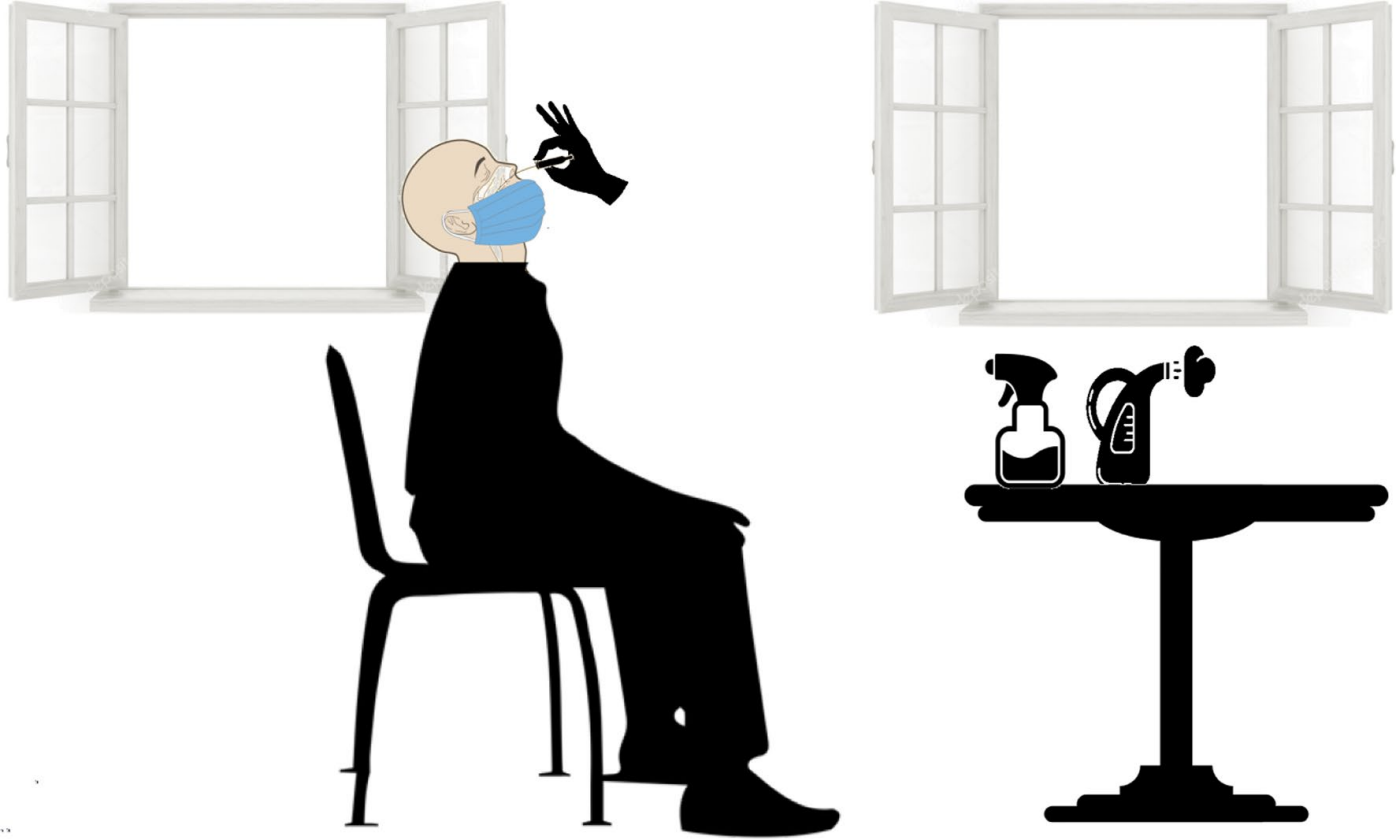
Proper sampling conditions for COVID-19 by nasopharyngeal swabs should provide good ventilation and light, washable surfaces and walls, readily available and safe decontaminants for ambient air and surfaces in the form of sprayer or steamer, while participants should be advised to keep their masks on their faces and only removing it from the nose area during the specimen collection to limit viral spreading by coughing or sneezing and to prevent breathing in aerosolized viral particles by only breathing through their mouth during the procedure. A careful swabbing technique can prevent most complications, even painfulness and sneezing. When inserting the swab along the nasal septum and bottom of common nasal meatus below the inferior turbinate, it is important never to touch the inferior turbinate.

The facemask of participants becomes of vital importance as it can limit both spreading and contacting the virus. Since the risk of contamination among frontline healthcare workers wearing a PPE is threefold higher than in the general population^27^, keeping the facemasks of participant on their mouths during the nasopharyngeal swabbing (Fig. 6) and standing sideways from the direction of potential infectious particles expelled by an AGE during the nasopharyngeal swabbing could limit the contamination of the examiner and the surroundings^28^.

#### Using disinfectants after an AGE

The use of chemical disinfectants or a UV lamp after an AGE can limit the contamination of the surrounding objects and the air. However, there are not many disinfectants that can be safely applied on objects and air with the participants inside, as their contact with the skin, eyes and respiratory mucosa can be harmful. 70% ethylic alcohol is an effective agent against enveloped viral pathogens as it acts on the integrity of the lipid bilayer membrane of the envelope. Methylene blue (MB) can be used as an adjuvant since it is safe for human use at low concentrations and it acts as a photosensitizer in association with visible light^29,30^ even for SARS-CoV2^31^.

#### Using the relative humidity of air after an AGE

Relative humidity (RH) of air is one of the most important factors affecting airborne virus infectivity as a direct link between the persistence, survival and pathogenesis of viral particles and RH was described^32^. Infectivity studies show that low RH tends to conserve the infectivity of enveloped viruses, while nonenveloped viruses become more stable at high RH^32^. It was shown that high RH is deleterious to the survival of the aerosolized enveloped coronaviruses SARS-CoV-2^3^, MERS-CoV^33^ and HCoV-229E^34^.

## Conclusions

The highly contagious SARS-CoV-2 persistent airborne viral aerosols produced by an infected person through sneezing and coughing represent a real threat to both the present room occupants or new occupants over the following hours from their emission time. The nasopharyngeal swab sample collection triggers AGEs in 1 in 5 participants to testing as our study shows. Given the changing dynamics of the viral spread, the chance of having AGEs from infected participants grows as the number of positive cases increases. Reducing the chances of contamination could be achieved using a well-designed specimen collection room with plenty of natural ventilation and light. Alternatively, collection of specimens in an outside non-fabric tent or through a drive-through approach are also possible alternatives. In addition, readily washable surfaces and an available sprayer or steamer with a harmless decontaminant for humans but potent antiviral activity should be used. Since the SARS-CoV-2 shows high resistance to multiple types of aggressions, the use o a combined strategy using both physical (heat, light, RH) and chemical (alcohol, MB photosensitizer) becomes justified. Requesting participants to keep their facemasks upon entry and only removing it from the nose area as the nasopharyngeal swab specimen is being collected while breathing only through the mouth, could limit both exposure to preexistent airborne viral particles and their generation by AGEs. Our study suggests that adequate PPE should be ensured for the testing personnel, while the development of additional strategies to limit the risk of contamination of other participants to the testing session from potentially infectious persistent aerosols should be implemented. Our findings represent arguments for implementing such measures and could contribute to a practice change in the current sampling method for the detection by nasopharyngeal swabbing of SARS-CoV-2 and other enveloped viral pathogens with airborne transmission.

## Data Availability

The datasets generated during and/or analysed during the current study are available from the corresponding author on reasonable request.
