# Author Correction: Assessment of the frequency of coughing and sneezing triggered by nasopharyngeal swabbing in the pandemic setting

**DOI:** 10.1038/s41598-022-16684-4

**Published:** 2022-07-18

**Authors:** Cosmin Andrei Cismaru, Sergiu Chira, Gabriel Laurentiu Cismaru, Andreea Mihaela Nutu, Mihai Gheorghe Netea, Ioana Berindan-Neagoe

**Affiliations:** 1 The Research Center for Functional Genomics, Biomedicine and Translational Medicine, “Iuliu Hatieganu” University of Medicine and Pharmacy, Cluj-Napoca, Romania; 2 Fifth Department of Internal Medicine, Cardiology-Rehabilitation, “Iuliu Hatieganu” University of Medicine and Pharmacy, Cluj-Napoca, Romania; 3 Department of Internal Medicine and Radboud Centre for Infectious Diseases, Radboud University Nijmegen Medical Center, 6525GA Nijmegen, The Netherlands

Correction to: *Scientific Reports* 10.1038/s41598-022-14755-0, published online 27 June 2022

The original version of this Article contained errors in the spelling of the authors Cosmin Andrei Cismaru, Sergiu Chira, Gabriel Laurentiu Cismaru, Andreea Mihaela Nutu, Mihai Gheorghe Netea and Ioana Berindan-Neagoe, which were incorrectly given as Cismaru Cosmin-Andrei, Chira Sergiu, Cismaru Gabriel-Laurentiu, Nutu Andreea-Mihaela, Netea Mihai-Gheorghe and Berindan-Neagoe Ioana.

The Author contribution statement now reads:

“C.A.C. performed the study concept and design. C.A.C., S.C. and A.M.N. performed the sampling and acquisition of data. G.L.C. performed the data analysis. All authors contributed to interpretation of data and revision of the report. I.B-N. and M.G.N. have been involved in drafting the manuscript and revising it critically for important intellectual content.”

The original Article has been corrected.

